# Machine-learning algorithm that can improve the diagnostic accuracy of septic arthritis of the knee

**DOI:** 10.1007/s00167-020-06418-2

**Published:** 2021-01-15

**Authors:** Eun-Seok Choi, Jae Ang Sim, Young Gon Na, Jong- Keun Seon, Hyun Dae Shin

**Affiliations:** 1 Department of Orthopaedic Surgery, Chungnam National University School of Medicine, Chungnam National University Hospital, 266 Munhwa-ro, Jung-gu, Daejeon, 35015 Republic of Korea; 2 Department of Orthopaedic Surgery, Gachon University College of Medicine, Gil Medical Centre, Incheon, Republic of Korea; 3 Department of Orthopaedic Surgery, CM Hospital, Seoul, Republic of Korea; 4 Department of Orthopaedic Surgery, Chonnam National University School of Medicine, Chonnam National University Hospital, Gwangju, Republic of Korea

**Keywords:** Machine learning, Infectious arthritis, Diagnosis, Knee

## Abstract

**Purpose:**

Prompt diagnosis and treatment of septic arthritis of the knee is crucial. Nevertheless, the quality of evidence for the diagnosis of septic arthritis is low. In this study, the authors developed a machine learning-based diagnostic algorithm for septic arthritis of the native knee using clinical data in an emergency department and validated its diagnostic accuracy.

**Methods:**

Patients (*n* = 326) who underwent synovial fluid analysis at the emergency department for suspected septic arthritis of the knee were enrolled. Septic arthritis was diagnosed in 164 of the patients (50.3%) using modified Newman criteria. Clinical characteristics of septic and inflammatory arthritis were compared. Area under the receiver-operating characteristic (ROC) curve (AUC) statistics was applied to evaluate the efficacy of each variable for the diagnosis of septic arthritis. The dataset was divided into independent training and test sets (comprising 80% and 20%, respectively, of the data). Supervised machine-learning techniques (random forest and eXtreme Gradient Boosting: XGBoost) were applied to develop a diagnostic model using the training dataset. The test dataset was subsequently used to validate the developed model. The ROC curves of the machine-learning model and each variable were compared.

**Results:**

Synovial white blood cell (WBC) count was significantly higher in septic arthritis than in inflammatory arthritis in the multivariate analysis (*P* = 0.001). In the ROC comparison analysis, synovial WBC count yielded a significantly higher AUC than all other single variables (*P* = 0.002). The diagnostic model using the XGBoost algorithm yielded a higher AUC (0.831, 95% confidence interval 0.751–0.923) than synovial WBC count (0.740, 95% confidence interval 0.684–0.791; *P* = 0.033). The developed algorithm was deployed as a free access web-based application (www.septicknee.com).

**Conclusion:**

The diagnosis of septic arthritis of the knee might be improved using a machine learning-based prediction model.

**Level of evidence:**

Diagnostic study Level III (Case–control study).

**Supplementary Information:**

The online version contains supplementary material available at 10.1007/s00167-020-06418-2.

## Introduction

The diagnosis of septic arthritis can be challenging [5]. No single serological marker has demonstrated sufficient sensitivity, specificity, or predictive value to distinguish septic arthritis from other types of arthritis [2, 7, 22]. Synovial fluid analysis has been considered to have potential for definitively diagnosing septic arthritis [5]. However, many studies have revealed that the predictive value of a single examination finding for septic arthritis may be weak [1, 2, 5, 14, 15]. The diagnosis of septic arthritis should be determined by the integration of a thorough history, physical examination, and the results of laboratory investigations [5, 16].

Machine learning has been applied to construct prediction models of complicated problems in which many factors are involved, while their respective relevance remains unclear. Hence, machine-learning algorithms have been applied in the field of knee surgery to predict events, such as postoperative acute kidney injury after total knee arthroplasty and the risk of hospital admission following anterior cruciate ligament reconstruction [10, 13].

Although many studies have concluded that comprehensive assessment is essential for the diagnosis of septic arthritis, what constitutes assessment in the clinical setting is ambiguous. The authors speculated that applying a machine-learning algorithm to the diagnosis of septic arthritis could be helpful for a comprehensive determination based on a variety of information and for immediate decision making in the emergency department (ED). To our knowledge, no study has been conducted with the aim of developing a diagnostic model of septic arthritis of the knee using a machine-learning algorithm. Therefore, the aim of the present study was to develop a machine learning-based diagnostic algorithm for septic arthritis of the native knee using clinical data in an ED and to validate its diagnostic accuracy.

## Materials and methods

### Patients

The authors conducted a retrospective study using data from two referral hospitals. Demographic and laboratory data, including data from serological testing and synovial fluid analysis, were collected from the electronic medical record system of each institution. Both institutions use a standardised medical record format for patients who visit the ED. Between January 2007 and December 2016, 866 adult patients visited the ED for the complaint of a painful swollen knee. To confine the dataset to septic arthritis of the native knee, 314 patients with a history of surgery (e.g. arthroplasty or arthroscopy) in the affected knee were excluded. Among the remaining 552 patients, 364 underwent surgical treatment based on the risk of septic arthritis of the knee. Among these 364 patients, 6 had incomplete medical records, and 12 had follow-ups of less than 1 year; consequently, these 18 patients were excluded. The authors enrolled 326 patients in the study. The demographic and clinical characteristics of the patients are shown in Table 1. This retrospective study was approved by our institutional review board. Informed patient consent was waived by the institutional review board.

**Table 1:** Comparison of clinical characteristics between septic arthritis group and inflammatory arthritis group

Characteristic	Septic arthritis (*n* = 164)	Inflammatory arthritis (*n* = 162)	Univariate	Multivariate
			*P*	*P*
Age (years)	67.1 (± 15.5)	63.6 (± 18.0)	0.060	
Sex			0.011*	0.322
Female	99 (57%)	74 (43%)		
Male	65 (42%)	88 (58%)		
Diabetes	35 (21.3%)	35 (21.3%)	0.424	
Body mass index	23.3 (± 3.4)	23.7 (± 3.4)	0.263	
Body temperature(°C)	37.1 (± 0.8)	37.4 (± 0.7)	0.774	
Serum				
Haemoglobin	11.8 (± 2.8)	12.0 (± 2.3)	0.756	
Hematocrit (%)	33.2 (± 4.7)	35.2 (± 6.5)	0.002*	0.408
White blood cell (× 10^9^/L)	10.6 (± 4.8)	9.7 (± 4.9)	0.089	
PMN count (%)	72.7 (± 10.3)	69.4 (± 11.3)	0.010*	0.724
Platelet (× 10^9^/L)	282.9 (± 113.8)	272.1 (± 108.8)	0.380	
Erythrocyte sedimentation rate	57.7 (± 29.5)	46.6 (± 32.9)	0.003*	0.544
C-reactive protein	13.1 (± 9.1)	10.2 (± 8.5)	0.003*	0.792
Uric acid*	**4.41 (± 1.92)**	**5.75 (± 3.84)**	0.001*	0.011*
Synovial fluid				
White blood cell (× 10^9^/L)*	**70.6 (± 75.4)**	**30.6 (± 31.6)**	< 0.001*	0.001*
PMN count (%)	88.7 (± 14.9)	81.4 (± 25.3)	< 0.001*	0.285
Presence of crystal	3 (0.6%)	17 (10.5%)	0.001*	0.143

Bold values with statistical significance level was defined as* P* < 0.05

*Statistically significant difference

### Diagnosis and treatment of septic arthritis

Among the types of data obtained in the ED, those features used to diagnose septic arthritis in previous studies were selected [2, 8, 12, 15]. Age, sex, body temperature, and the presence of chronic diseases (diabetes or hypertension) were investigated. In serological tests, complete blood cell counts and levels of inflammatory markers (CRP and ESR) were determined. Synovial fluid metrics, including cell counts, were identified. Decisions regarding surgery were made by senior orthopaedic surgeons based on the clinical presentation and laboratory test results. Arthroscopic irrigation and synovectomy were performed for all patients within 24 h following the initial visit. The authors applied Newman criteria [16] for case definition of septic arthritis. One of the following conditions had to be met to make the diagnosis: (1) isolation of a pathogenic organism from the affected joint; (2) isolation of a pathogenic organism from another source (e.g. blood) in the context of a hot red joint suspicious of sepsis; (3) typical clinical features and turbid joint fluid in the presence of previous antibiotic treatment; and (4) postmortem or pathological features suggestive of septic arthritis. Senior orthopaedic surgeons who performed arthroscopic surgery evaluated the criteria and confirmed the diagnosis.

### Machine-learning procedure

In machine learning, a model learns to distinguish patterns in data [20]. In the present study, the authors used supervised learning, in which the learning occurs from examples provided in the form of inputs (called features) and outputs (called labels). In this study, the authors developed a binary classifier model that predicts the possibility of septic arthritis using the above-described 16 features obtained from the ED. The models were constructed using Python.

The raw data were preprocessed with the scikit-learn library to produce a scaled and normalised dataset. To address the missing values in the training set, the authors applied an imputation algorithm based on the k-nearest neighbour method in the scikit-learn library. Categorical variables were converted to numerical data using the one-hot encoder algorithm. The set of processed datasets was randomly divided into two sets. The first, the training set (comprising 80% of the entire dataset), was used to build the model. The remaining 20% was used as a test set to assess the prediction accuracy of the model. For hyperparameter tuning, optimal hyperparameter was applied using GridSearch from the scikit-learn library. When an algorithm is too optimised for a single set of training data, it can memorise the data rather than learning to generalise from the data. Hence, its prediction accuracy may be lowered (i.e. it may overfit the data). The authors performed fivefold cross-validation of the training dataset to obtain the optimal degree of model complexity to avoid overfitting. In the fivefold cross-validation, the dataset was randomly partitioned five times, with a different partition chosen each time. The training accuracy was evaluated by the mean area under the curve (AUC) value of the receiver-operating characteristic (ROC). To understand the decision-making process, the authors needed to identify the importance of each feature in the machine-learning algorithm. Therefore, the authors calculated the importance of each feature.

Extreme Gradient Boosting (XGBoost) and random forest (RF) algorithms were used to predict the probability of septic arthritis. The XGBoost algorithm is an extendible gradient boosting machine that can be applied to both regression and classification [6]. Since its introduction in 2014, the impact of XGBoost has been widely recognised in several machine learning and data-mining challenges. Recently, it has been widely used for the classification and prediction of medical problems [19, 23]. The RF algorithm is a commonly used decision tree-based method with random elements. RFs iterate over many boosted datasets and create many different training models to form an ensemble model. RFs have been applied to various medical problems to predict mortality or to analyse gene expression data [11, 18].

The final models of each algorithm were applied to the independent test dataset to evaluate the probabilities and compare discriminatory power. The authors performed validation using the test dataset and evaluation using the ROC with the AUC. The authors deployed the optimal algorithm as a web-based application (www.septicknee.com). When the 16 features used in this study are inputted into the algorithm, the probability of septic arthritis is expressed in %, and the importance of each feature used in the decision making is displayed in a graph.

### Statistical analysis

The authors used the independent *t* test for continuous variables, Pearson’s Chi-square for categorical variables and a linear regression model for multivariate analysis. The odds ratio of each variable was calculated. The ROC with AUC according to the clinical infection was used to evaluate model sensitivity and specificity. The method of DeLong et al. was applied to compare the ROC curves [4]. The significance of differences in AUC between each variable and each machine-learning algorithm was analysed using MedCalc (Ostend, Belgium, MedCalc Software Ltd.). The statistical significance level was defined as *P* < 0.05. Other statistical analyses were performed using SPSS (version 15, Chicago, SPSS Inc.).

## Results

Of the 326 patients, 164 (50.3%) were defined as having septic arthritis according to modified criteria described by Newman. The clinical characteristics of septic and inflammatory arthritis are shown in Table 1. Serum uric acid level and synovial WBC count showed significant differences between septic and inflammatory arthritis in the multivariate analysis (*P* = 0.011 and *P* = 0.001, respectively). The odds ratios of serum uric acid level and synovial WBC count were 0.816 and 1.231, respectively. Among the features, synovial WBC count yielded the highest AUC (0.740), followed by ESR level, synovial polymorphonuclear leukocyte (PMN) level, CRP level, and serum PMN level (Table 2). Other continuous variables yielded AUC values under 0.500. The optimal cutoff value of synovial WBC count according to the ROC analysis was 27.4 × 10^9^/L. When the cutoff value of synovial WBC count was set to 50.0 × 10^9^/L, the sensitivity and specificity for septic arthritis were 53.7% and 82.7%, respectively.

**Table 2:** Comparison of area under the curve (AUC) for single variables

Variable	AUC	95% Confidence interval	Cut-off value	Sensitivity	Specificity
Serum WBC count (× 10^9^/L)	0.541	0.480–0.601	12.1	31.3	80.1
Serum uric acid	0.542	0.471–0.612	3.9	64.8	48.9
C-reactive protein	0.573	0.513–0.632	14.2	40.5	76.9
Synovial PMN neutrophils (%)	0.589	0.529–0.648	89.5%	72.8	42.6
Erythrocyte sedimentation rate (mm/h)	0.610	0.550–0.668	24.0	85.5	33.1
Synovial WBC count (× 10^9^/L)	0.740	0.684–0.791	27.4	81.1	59.3

Synovial WBC counts showed statistically significantly higher AUC values than all other single variables (*P* = 0.002)

### Accuracy of the machine-learning models

The AUC values of the RF and XGBoost algorithms using the training dataset were 0.912 (95% CI 0.879–0.947) and 0.927 (95% CI 0.884–0.954), respectively. The verified AUC of the RF and XGBoost algorithms using the independent test dataset were 0.801 (95% CI 0.699–0.907) and 0.831 (95% CI 0.754–0.914), respectively. In the ROC comparison analysis, synovial WBC count yielded a significantly higher AUC value than all of the other single variables (*P* = 0.002). However, the XGBoost algorithm yielded a significantly higher AUC value than the RF algorithm and synovial WBC count (*P* = 0.001 and *P* = 0.033, respectively).

### Importance of the features

**Fig. 1:**
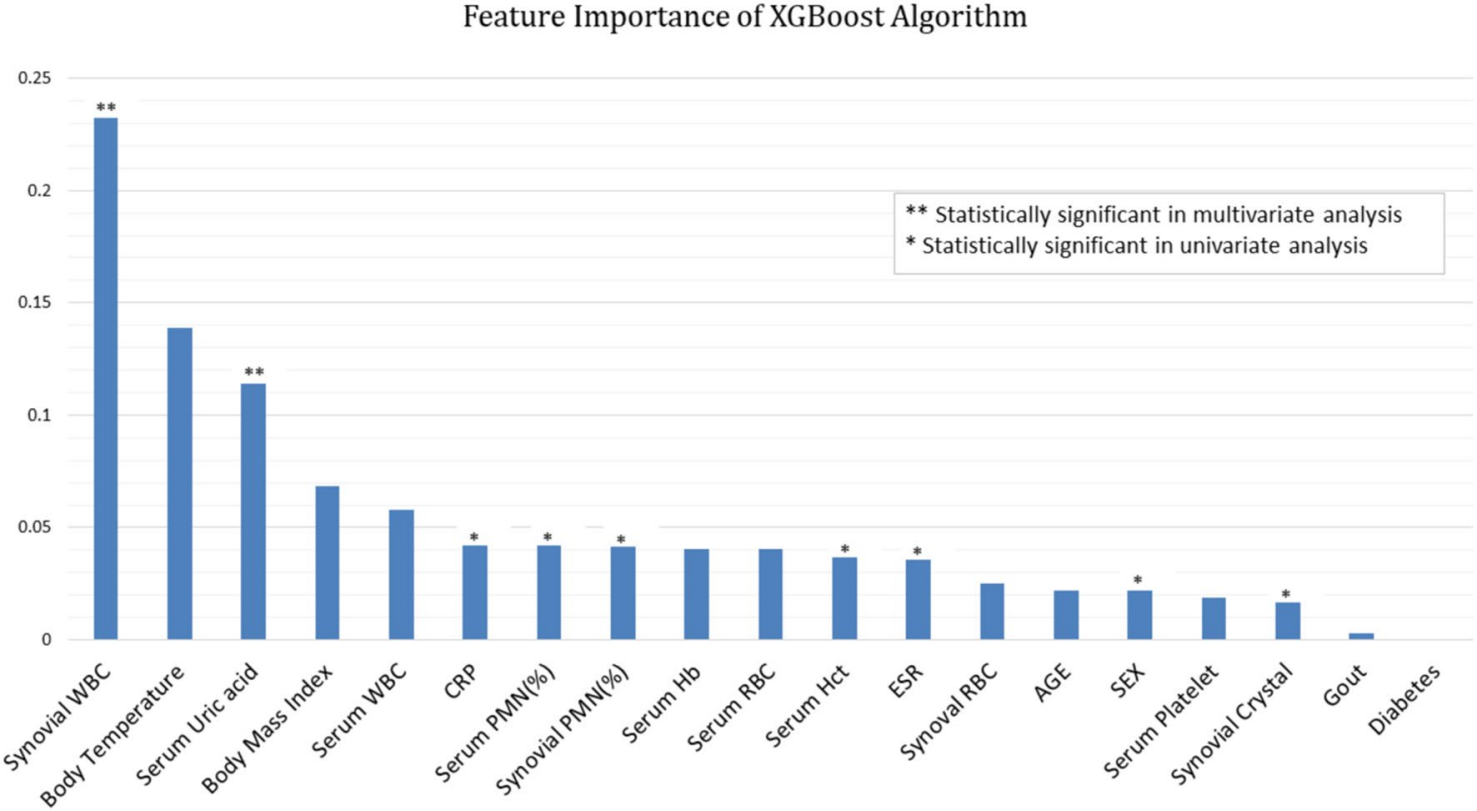
Feature importance of the XGBoost algorithm. Features that showed significance in multivariate analysis also ranked high in importance in the XGBoost algorithm

For the XGBoost algorithm, the authors evaluated the importance ranks, which indicate the importance of the input features. As shown by the linear regression model, synovial WBC was the most important feature in the decision process (feature importance: 0.232) (Fig. 1). Serum uric acid level had the third-highest importance (0.114). Features that showed significance in only the univariate analysis had less than 0.05 significance. In the RF algorithm, synovial WBC count was the most important feature in the decision process (feature importance: 0.275). The order of feature importance for the RF algorithm was synovial WBC count > body temperature > and serum uric acid level. However, the order and importance of the remaining features differed from those of the XGBoost algorithm.

## Discussion

The most important finding of the present study was that the novel approach using a machine learning-based diagnostic model of septic arthritis of the native knee showed superior accuracy compared to the traditional method of using a single variable.

No single clinical sign or laboratory value alone is conclusive for diagnosing septic arthritis [3]. Synovial WBC count has been considered an important value for the definite diagnosis of septic arthritis [5]. Consistent with the results of the present study, a meta-analysis of 32 studies regarding the diagnosis of septic arthritis confirmed that only extreme values of synovial WBC (> 50 × 10^9^/L) are specific but not sensitive for the diagnosis of septic arthritis [2]. In adult septic arthritis of the knee, validated criteria reflecting independent predictors have not been proposed.

In clinical situations in which several variables need to be comprehensively assessed, as in the diagnosis of septic arthritis of the knee, machine-learning algorithms have promise as a new approach. Several studies have revealed machine-learning algorithms to be superior to logistic regression at predicting perioperative risks of arthroplasty or patient-reported outcome measures after arthroscopy [10, 13, 17]. The authors of the present study obtained higher prediction accuracy using the XGBoost algorithm than synovial WBC count. The XGBoost algorithm used in this study calculates the importance of each feature using a repeated learning process without dropping out the input features. Each feature participates in the decision process according to its determined importance.

It is important that orthopaedic surgeons be made aware of the benefits of the machine-learning algorithms. A common concern regarding the use of machine-learning algorithms in practice is their “black-box” nature. Since the gradient boost algorithms, including XGBoost, are based on the decision tree method, they are not “black-box” algorithms in the same way as deep learning algorithms. The results of these tree ensemble models can be easily explained and interpreted when the model has a small number of trees and each tree has a shallow depth. Importance scores indicate how valuable a feature is in the construction of boosted trees within the model. In recent studies applying the gradient boost algorithm, the authors tried to infer the decision-making process by referring to the feature importance applied to the tree decision [9, 10]. Synovial WBC count and serum uric acid level, which were significant in the multivariate analysis (Table 1), also showed high importance in the XGBoost algorithm (Fig. 1). This finding suggests that the feature importance of the machine-learning algorithm was not significantly different from that indicated by existing statistical methods. The authors have deployed the developed algorithm as a free access web-based application (www.septicknee.com). The probability of septic arthritis, the accuracy of the prediction, and the importance of the features are reported in the application.

## Limitations

Since this study is retrospective in design, it might have selection bias. The authors included only patients with a definitive diagnosis of septic arthritis that had been confirmed through surgery. This inclusion process might have biased the study population towards those with a higher suspicion of septic arthritis and excluded those with a lower suspicion. The population sampled in this study might not represent all patients who visit the ED with an acute swollen knee. Additional studies are needed to verify the diagnostic algorithm through a prospective study regardless of the level of suspicion of septic arthritis of the knee.

Definitive diagnosis of septic arthritis is challenging. If bacteria are isolated from the synovial fluid, the diagnosis of septic arthritis is definitive. However, the absence of organisms on Gram stain or a negative synovial fluid culture can occur with sensitive organisms or previous antibiotic treatment. Such results do not exclude the diagnosis [15]. Blood cultures are positive in only approximately 50% of cases. Synovial fluid culture is only 75% sensitive for the diagnosis of septic arthritis [21]. The authors applied Newman criteria for the case definition of septic arthritis, which have been applied to classify sterile culture conditions [16].

This study was based on information from the ED. Since the medical history was confirmed depending on the patient’s response, only the presence or absence of a limited disease was confirmed. The authors have not investigated all conditions that could affect inflammatory markers or synovial fluid analysis, such as rheumatoid arthritis.

A small amount of the training dataset may affect the performance and robustness of the developed algorithm. In the present study, the authors developed and validated an algorithm with data from 80% of all patients used as the training set and data from the remaining 20% used as the test set. The 326 refined datasets used in this study represent a large number relative to those of previous studies. Nevertheless, more data are needed to improve the accuracy of the algorithm.

The proposed machine-learning algorithm may facilitate holistic analysis of clinical findings and laboratory values. A web-based application was deployed to support the accurate diagnosis of septic arthritis, and the probability and feature importance can be immediately confirmed.

## Conclusion

The machine learning-based diagnostic algorithm for septic arthritis of the native knee demonstrated higher diagnostic accuracy than any other single variable. The diagnostic accuracy of the septic arthritis of the knee might be improved using machine-learning prediction models.

## Supplementary Information

Below is the link to the electronic supplementary material.

Supplementary file1 (PDF 69 KB)

Supplementary file2 (CSV 25 KB)

## Abbreviations

AUC: Area under the receiver-operating characteristic curve
ROC: Receiver-operating characteristic
WBC: White blood cell
XGBoost: eXtreme Gradient Boosting
ESR: Erythrocyte sedimentation rate
CRP: C-reactive protein concentration
PMN: Polymorphonuclear leukocyte
ED: Emergency department
RF: Random forest
