# Supplementary material for: Machine-learning algorithm that can improve the diagnostic accuracy of septic arthritis of the knee
Source: Knee Surg Sports Traumatol Arthrosc. 2021 Jan 15;29(10):3142–8. doi: 10.1007/s00167-020-06418-2 (PMC8458173; doi:10.1007/s00167-020-06418-2)
Supplement: Supplementary file 1 — Supplementary file1 (PDF 69 KB) [file 167_2020_6418_MOESM1_ESM.pdf]

```python
import copy
import warnings
import gc
warnings.filterwarnings("ignore")
gc.enable()

import scipy
import numpy as np
import pandas as pd
import xgboost as xgb
import lightgbm as lgb
from xgboost import plot_importance as xgb_plot_importance
from fancyimpute.iterative_imputer import IterativeImputer

import sklearn
from sklearn.ensemble import RandomForestClassifier, GradientBoostingClassifier, AdaBoostClassifier
from sklearn.metrics import accuracy_score, precision_score, roc_auc_score, confusion_matrix, f1_score
from sklearn.model_selection import train_test_split, StratifiedKFold, cross_val_score
from sklearn.utils import shuffle

from imblearn.metrics import specificity_score, sensitivity_score

import tensorflow as tf
from keras import backend as K
```

```
import keras
from keras import layers, models, Input


import seaborn as sns
import matplotlib.pyplot as plt
plt.style.use('fivethirtyeight')
pd.set_option('max_rows', 500)
pd.set_option('max_colwidth', 500)
pd.set_option('max_columns', 500)
data = pd.read_csv('SepticKnee_raw2.csv')
data.shape
sns.heatmap(data.corr(),annot=True,cmap='RdYlGn',linewidths=0.8)
fig=plt.gcf()
fig.set_size_inches(16,10)
plt.show()
positive = data[data['Clinical_infection'] == 1]
negative = data[data['Clinical_infection'] == 0]
labels = ['Positive', 'Negative']
values = [len(positive), len(negative)]
explode = [0.1, 0]
plt.pie(x=values,
        explode=explode,
        autopct='%1.1f%%',
        labels=labels,
        shadow=True
       )
```

```
plt.show()
for c in positive.columns[1:] :
    plt.hist(positive[~positive[c].isna()][c], alpha=0.5, bins=30)
    plt.hist(negative[~negative[c].isna()][c], alpha=0.5, bins=30)
    plt.legend(['positive', 'negative'])
    plt.title('Column : {}'.format(c))
    plt.show()
from sklearn.preprocessing import StandardScaler


def impute(data, target, train=False):
    global col_list
    data = copy.deepcopy(data)
    data['Clinical_infection'] = target
    #Split by Target
    positive = copy.deepcopy(data[data['Clinical_infection'] == 1])
    negative = copy.deepcopy(data[data['Clinical_infection'] == 0])

    #BMI
    positive['BMI'].fillna(positive['BMI'].median(), inplace=True)
    negative['BMI'].fillna(negative['BMI'].median(), inplace=True)

    #others
    imputer = IterativeImputer(n_iter = 20, random_state=42)
    imputed_positive = pd.DataFrame(imputer.fit_transform(positive[col_list]), columns=col_list)
    imputed_negative = pd.DataFrame(imputer.fit_transform(negative[col_list]), columns=col_list)
    imputed_positive['Clinical_infection'] = positive['Clinical_infection'].values
```

```
        imputed_negative['Clinical_infection'] = negative['Clinical_infection'].values


        data = shuffle(pd.concat([imputed_positive, imputed_negative]), random_state=42)
    if train :
            noised_data = copy.deepcopy(data)
            for c in continuous_columns:
                noised_data[c] = noised_data[c] + np.random.normal(size=noised_data[c].shape)
            data = pd.concat([data, noised_data])
    return data[col_list], data['Clinical_infection']


x = copy.deepcopy(data)
y = copy.deepcopy(data['Clinical_infection'])


col_list = [c for c in positive if c not in ['Clinical_infection']]


del x['Clinical_infection']
x_train, x_test, y_train, y_test = train_test_split(x, y, test_size = 0.2, stratify=y, random_state=2019)
folds = StratifiedKFold(5, random_state=2019)
xgb_params = {
                'lambda': 0.01099,
                'eta': 0.17947,
                'min_child_weight': 1.09265,
                'booster': 'gbtree',
                'alpha':0.11773,
                'max_depth': 2,
                'colsample_bytree': 0.62456,
```

```
                    'gamma': 0.33170,
                    'learning_rate' : 0.01,
                    'subsample': 0.6,
                    'objective': 'binary:logistic',
                    'eval_metric': 'auc',
                    'seed': 2019,
                    'silent': 1,
                }
from sklearn.metrics import roc_curve
def draw_roc(y_test, y_score, label):
    fpr, tpr, _ = roc_curve(y_test, y_score)
    plt.plot([0, 1], [0, 1], 'k--')
    plt.plot(fpr, tpr, label=label)
    plt.xlabel('False positive rate')
    plt.ylabel('True positive rate')
    plt.title(label+' ROC curve')
    plt.legend(loc='best')
    plt.show()
def CrossValwithEnsemble(clf, x_train, y_train, x_test, y_test):
    auc_list = list()
    oof_list = list()
    # validation
    global oof_preds, test_preds, imputed_y_test
    num_round = 20000
    oof_preds = np.zeros(y_train.shape)
    fold_by_oof = np.zeros((folds.n_splits, y_train.shape[0]))
```

```
# test results
test_preds = np.zeros(y_test.shape)
fold_by_test = np.zeros((folds.n_splits, y_test.shape[0]))
feature_importance_df_deep = pd.DataFrame()
y_train_valid_label = np.zeros(y_train.shape)

imputed_x_test, imputed_y_test = impute(x_test, y_test)

if clf == xgb:
    params = xgb_params
else :
    params = lgb_params

for n_fold, (trn_idx, val_idx) in enumerate(folds.split(x_train, y_train)):
    trn_x, trn_y = impute(x_train.iloc[trn_idx], y_train.iloc[trn_idx], train=True)
    val_x, val_y = impute(x_train.iloc[val_idx], y_train.iloc[val_idx])
    y_train_valid_label[val_idx] = val_y

    if clf == xgb:
        label = 'XGboost'
        train_data = clf.DMatrix(data=trn_x, label=trn_y)
        valid_data = clf.DMatrix(data=val_x, label=val_y)
        watchlist = [(train_data, 'train'), (valid_data, 'valid')]
        print("xgb " + str(n_fold) + "-" * 50)
```

```
                model = xgb.train(params, train_data, num_round, watchlist,
early_stopping_rounds=200, verbose_eval=400)


                oof_preds[val_idx] = model.predict(clf.DMatrix(val_x),
ntree_limit=model.best_ntree_limit+50)

                test_preds += model.predict(clf.DMatrix(imputed_x_test),
ntree_limit=model.best_ntree_limit+50) / (folds.n_splits)


                fold_by_oof[n_fold, val_idx] = model.predict(clf.DMatrix(val_x),
ntree_limit=model.best_ntree_limit+50)

                fold_by_test[n_fold] = model.predict(clf.DMatrix(imputed_x_test),
ntree_limit=model.best_ntree_limit+50)


    # Feature Importance

    try :

        if clf == xgb:

            trn_x, trn_y = impute(x_train, y_train)

            tst_x, tst_y = impute(x_test, y_test)


            train_data = clf.DMatrix(trn_x, label=trn_y)

            valid_data = clf.DMatrix(tst_x, label=tst_y)

            watchlist = [(train_data, 'train'), (valid_data, 'valid')]


            model = clf.train(params, train_data, num_round, watchlist,
```

```
early_stopping_rounds=200, verbose_eval=400)
                xgb_plot_importance(model)
            else :
                trn_x, trn_y = impute(x_train, y_train)
                tst_x, tst_y = impute(x_test, y_test)

                train_data = clf.Dataset(trn_x, label=trn_y)
                valid_data = clf.Dataset(tst_x, label=tst_y)
                watchlist = [train_data, valid_data]

                model = clf.train(params, train_data, num_round, watchlist,
early_stopping_rounds=200, verbose_eval=400)
                lgb_plot_importance(model)
        except :
            if clf != keras:
                trn_x, trn_y = impute(x_train, y_train)
                tst_x, tst_y = impute(x_test, y_test)
                model.fit(trn_x, trn_y)

            if clf == RandomForestClassifier or clf == AdaBoostClassifier or
clf==GradientBoostingClassifier:
                importances = model.feature_importances_
                indices = np.argsort(importances)
```

```
            plt.title('Feature Importances')
            plt.barh(range(len(indices)), importances[indices], color='b', align='center')
            plt.yticks(range(len(indices)), [x_train.columns[i] for i in indices])
            plt.xlabel('Relative Importance')
            plt.show()

    val_list = list()
    test_list = list()
    for i in range(folds.n_splits):
        test_list.append(roc_auc_score(imputed_y_test.values, fold_by_test[i]))

    draw_roc(imputed_y_test.values, test_preds, label)
    cv_auc = roc_auc_score(y_train_valid_label, oof_preds)
    test_auc = roc_auc_score(imputed_y_test.values, test_preds)

    print("CV AUC: {:.2f}".format(cv_auc))
    print("Test CV AUC: {:.2f}[{:.2f}, {:.2f}]₩n₩n".format(test_auc, test_auc- 1.96*test_auc_std,
1.96*test_auc_std + test_auc))
    return evaluate(imputed_y_test, test_preds)
def evaluate(imputed_y_test, test_preds):
    conf_mat = pd.DataFrame(confusion_matrix(imputed_y_test, [ 1 if x > 0.5 else 0 for x in
test_preds]), columns = ['pred_negative', 'pred_positive'], index=['negative', 'positive'])

    threshold = np.linspace(0, 1, 101)
```

```
pred_list = list()
for t in threshold :
    temp = list()

    y_pred = list()
    for x in test_preds:
        if x >= t :
            y_pred.append(1)
        else:
            y_pred.append(0)

    temp.append(accuracy_score(imputed_y_test.values, y_pred))
    temp.append(sensitivity_score(imputed_y_test.values, y_pred))
    temp.append(specificity_score(imputed_y_test.values, y_pred))
    temp.append(precision_score(imputed_y_test.values, y_pred))
    temp.append(f1_score(imputed_y_test.values, y_pred))

    pred_list.append(temp)

pred_list = np.array(pred_list)

result_df = pd.DataFrame(pred_list, columns =['Accuracy', 'Sensitivity', 'Specificity', 'Precision', 'F1_score'], index=threshold)
```

```
return result_df
```
